# E/E′ Is a New Independent Predictor of Recovered Ejection Fraction in Patients With Systolic Heart Failure Undergoing Ablation for Atrial Fibrillation

**DOI:** 10.3389/fcvm.2021.707996

**Published:** 2022-01-13

**Authors:** Minghui Yang, Rongfeng Zhang, Huamin Tang, Guocao Li, Xumin Guan, Yiheng Yang, Yuanjun Sun, Xianjie Xiao, Xiaohong Yu, Xiaomeng Yin, Yingxue Dong, Lianjun Gao, Yunlong Xia, Yanzong Yang

**Affiliations:** First Affiliated Hospital, Dalian Medical University, Dalian, China

**Keywords:** atrial fibrillation, E/E′, recovered ejection fraction, heart failure, catheter ablation

## Abstract

**Aims:** Catheter ablation should be considered in patients with atrial fibrillation (AF) and with heart failure (HF) with reduced ejection fraction (EF; HFrEF) to improve survival and reduce heart failure hospitalization. Careful patient selection for AF ablation is key to achieving similar outcome benefits. However, limited data exist regarding predictors of recovered ejection fraction. We aimed to evaluate the predictors of recovered ejection fraction in consecutive patients with HF undergoing AF ablation.

**Methods and Results:** A total of 156 patients [67.3% men, median age 63 (11)] with AF and HF underwent initial catheter ablation between September 2017 and October 2019 in the First Affiliated Hospital of Dalian Medical University. Overall, the percentage of recovered ejection fractions was 72.3%. Recovered EFs were associated with a 39% reduction in all-cause hospitalization compared to non-recovered EFs at the 1-year follow-up [23.8 vs. 62.8 (odds ratio) OR 2.09 (1.40–3.12), *P* < 0.001]. Univariate analysis for recovered EFs showed that diabetes (*P* = 0.083), prevalent HF (*P* = 0.014), prevalent AF (*P* = 0.051), LVEF (*P* = 0.022), and E/E′ (*P* = 0.001) were associated with EF improvement. Multivariate analysis showed that the only independent predictor of EF recovery was E/E′ [OR 1.13 (1.03–1.24); *P* = 0.011]. A receiver operating characteristic analysis determined that the suitable cut-off value for E/E′ was 15 (sensitivity 38.7%, specificity 89.2%, the area under curve 0.704).

**Conclusions:** Ejection fraction (EF) recovery occurred in 72.3% of patients, associated with a 39% reduction in all-cause hospitalization compared to the non-recovered EFs in our cohort. The only independent predictor of recovered EF was E/E′ < 15 in our series.

## Introduction

The atrial fibrillation epidemic has been closely linked to a concomitant rise in heart failure (HF) morbidity and mortality (1). The estimated incidence of HF among patients with atrial fibrillation (AF) is 1.58 to 4.4 per 100 person-years. HF and AF often coexist in clinical practice (2). When present in combination, AF and HF portend a worse prognosis than either condition alone.

Recent randomized controlled trials (RCTs) reported clinical improvements in mortality, HF hospitalizations, left ventricular ejection fraction (LVEF), and quality of life in patients with HFrEF (HF with reduced ejection fraction) who had AF ablation (3–5). The Catheter Ablation vs. Medical Rate Control in AF and Systolic Dysfunction (CAMERA-MRI) and Pulmonary-Vein Isolation for AF Patients with HF (PABACHF) trials reported that 58–76% of patients had normalization of EF after AF ablation compared with patients receiving another medical therapy (6, 7). CASTLE-AF was the largest RCT to compare the hard endpoints between ablation and medical therapy in patients with AF and HF (8). Catheter Ablation versus Standard Conventional Therapy in Patients with Left Ventricular Dysfunction and Atrial Fibrillation (CASTLE-AF) revealed a benefit of mortality and HF hospitalizations in AF ablation patients. However, only a small number of selected patients underwent AF ablation in these trials, the largest one having only 179 patients randomized to the AF ablation group. The AF Management in Congestive HF with Ablation (AMICA) trial was a large RCT to compare the absolute increase in LVEF from baseline at 1 year between ablation and the best medical therapy in patients with persistent AF and HF (9). The AMICA trial did not reveal any benefit of AF ablation in patients with AF and advanced HF. These controversial results raised the issue that stratification for AF in HF patients remains challenging in clinical practice. Patients with HF and AF benefit the most from catheter ablation should be fully evaluated.

In this study, we carried out a retrospective study to evaluate predictors of LVEF recurrence after ablation for AF in systolic HF patients.

## Methods

### Patient Selection

The protocol was reviewed and approved by the ethics committee of the First Affiliated Hospital of Dalian Medical University. Patients presenting with a documented episode of symptomatic AF and systolic HF (LVEF < 50%) were eligible for enrollment in the study. Potential clinical predictors were analyzed, including age, sex, type of AF, CHA2DS2-VASc score, complications, echocardiogram characteristics, and health history.

Atrial fibrillation (AF) classification was defined as:

*Paroxysmal AF*: Self-terminating, in most cases within 48 h. Some AF paroxysms may continue for up to 7 days. AF episodes that are cardioverted within 7 days should be considered paroxysmal.

*Persistent AF*: AF that lasts longer than 7 days, including episodes terminated by cardioversion, either with drugs or by direct current cardioversion, after 7 days or more.

#### Catheter Ablation Strategy

The indications for AF ablation procedures and periprocedural anticoagulation were in accordance with the current guidelines. Preprocedural left atrial CT was performed to evaluate the anatomy of the pulmonary veins. Immediately before the ablation procedure, the presence of a left atrial appendage thrombus was excluded with transesophageal echocardiography. In patients undergoing catheter ablation, circumferential PV isolation was mandatory as the primary method. Additional ablation techniques, including the creation of linear lesions, ablation of complex fractionated atrial electrograms, or combinations thereof, were left to the investigator's discretion for a secondary ablative approach. The endpoint of the ablation strategy was complications of the PVI and/or additional lesions. Mapping was performed with the Carto 3D cardiac mapping system (CARTO, Biosense Webster, Inc., US).

#### Recovered Ejection Fraction

A working definition of RecEF that is consistent with the majority of studies in the literature includes the following: (1) Documentation of a decreased LVEF < 40% at baseline; > 10% absolute improvement in LVEF and the second measurement of LVEF > 40% (10). (2) Documentation of a decreased LVEF 40–50% at baseline and the second measurement of LVEF > 50%. (3) LVEF measurements were obtained under sinus rhythm (SR) or AF at baseline (30.8% under SR, 69.2% under AF). After ablation, a second measurement was obtained at sinus rhythm (78.2% under SR, 21.8% under AF).

#### Follow-Up and Echocardiograms

For the whole 1-years follow-up (FU), all patients were monitored in the out-patient department of our institution. Oral anticoagulation was uninterrupted during the follow-up. A designated follow-up clinic completed the follow-ups. Patients had follow-ups in the postoperative months at 1, 3, 6, and 12 *via* clinic visit. ECG or 24-h Holter and echocardiograms were obtained at the clinic visit. Echocardiograms were centrally assessed at our echocardiography laboratory. LVEF assessment was originally intended to use contrast echocardiography at sinus rhythm. All patients underwent standardized, 2D non-contract transthoracic echocardiographic imaging. LVEF was determined according to the Simpson rule from left ventricular end-diastolic and end-systolic volumes in apical 5- and 2-chamber views. All causes of death and hospitalization were obtained at 12 months.

### Statistics

Baseline characteristics were summarized as means and *SD*s for continuous variables or frequency numbers and percentages for categorical variables. Differences between the two groups were estimated with 2-sample *t*-tests for continuous variables or chi-square analyses for categorical variables. Logistics proportional hazards models were used to adjust for differences in baseline characteristics or pertinent covariates on outcomes. We estimated univariable and multivariable models, hazard ratios (HRs), and their relative 95% CIs were derived. Covariates selected for multivariable models were based on significant variables in the univariable analyses and entered into models stepwise. The multivariate analysis and logistic regression were conducted to evaluate the predictors. A two-tailed *P* < 0.05 was considered significant. All statistical analyses were performed using IBM SPSS Statistics software version 24 (SPSS Inc., IBM, Somers, New York, USA).

## Results

### Characteristics of Enrolled Patients

During the 3 years, 156 patients with AF and systolic HF (LVEF < 50%) were enrolled in the study. The median age was 64 years (53–69 years), and 33% were females. The average LVEF was 37.9%. Baseline characteristics for the study population are shown in Table 1.

**Table 1:** Characteristics of patients at baseline.

	***n* = 156**
Age, mean (SD), years	63.7 (11.0)
**Sex, no. (%)**	
Male	67.3%
Female	32.7%
**Type of AF, no. (%)**	
Paroxysmal	30.8%
Persistent	65.4%
Long-standing	4.8%
Duration of AF, mean (SD), years	3.3 (5.0)
Hypertension, no. (%)	73 (46.8)
Post MI, no (%)	16 (10.3)
Dilated cardiomyopathy, no. (%)	5 (3.2)
Hypertrophic cardiomyopathy, no. (%)	1 (0.6)
Ischaemic cardiomyopathy, no. (%)	11 (7.1)
Stroke, transient ischaemic attack, peripheral embolism, no. (%)	16 (10.9)
Diabetes, no. (%)	28 (17.9)
Coronary heart disease, no. (%)	21 (13.5)
Valvular heart disease, no. (%)	16 (10.3)
CHA_2_DS_2_-VASc score, mean (SD)	2.37 (1.45)
**MYHA class, no. (%)**	
I	28 (17.9)
II	52 (33.3)
III	62 (39.7)
IV	14 (9.0)
LVEF, mean (SD), %	37.9 (7.8)
NT-proBNP, mean (SD), pg/ml	640 (934.8)
TnI, mean (SD), ng/ml	0.06 (0.17)
D-dimer, mean (SD), mg/l	0.61 (1.26)
Oral anticoagulation (%)	152 (97.4)
β blocker used (%)	66 (42.3)
ARNI used (%)	71 (45.2)
ACEI/ARB used (%)	67 (42.9)

*AF, atrial fibrillation; LVEF, left ventricular ejection fraction; MI, myocardial infarction*.

### Recovery and Outcome of Heart Failure After Atrial Fibrillation Ablation

**Figure 1:**
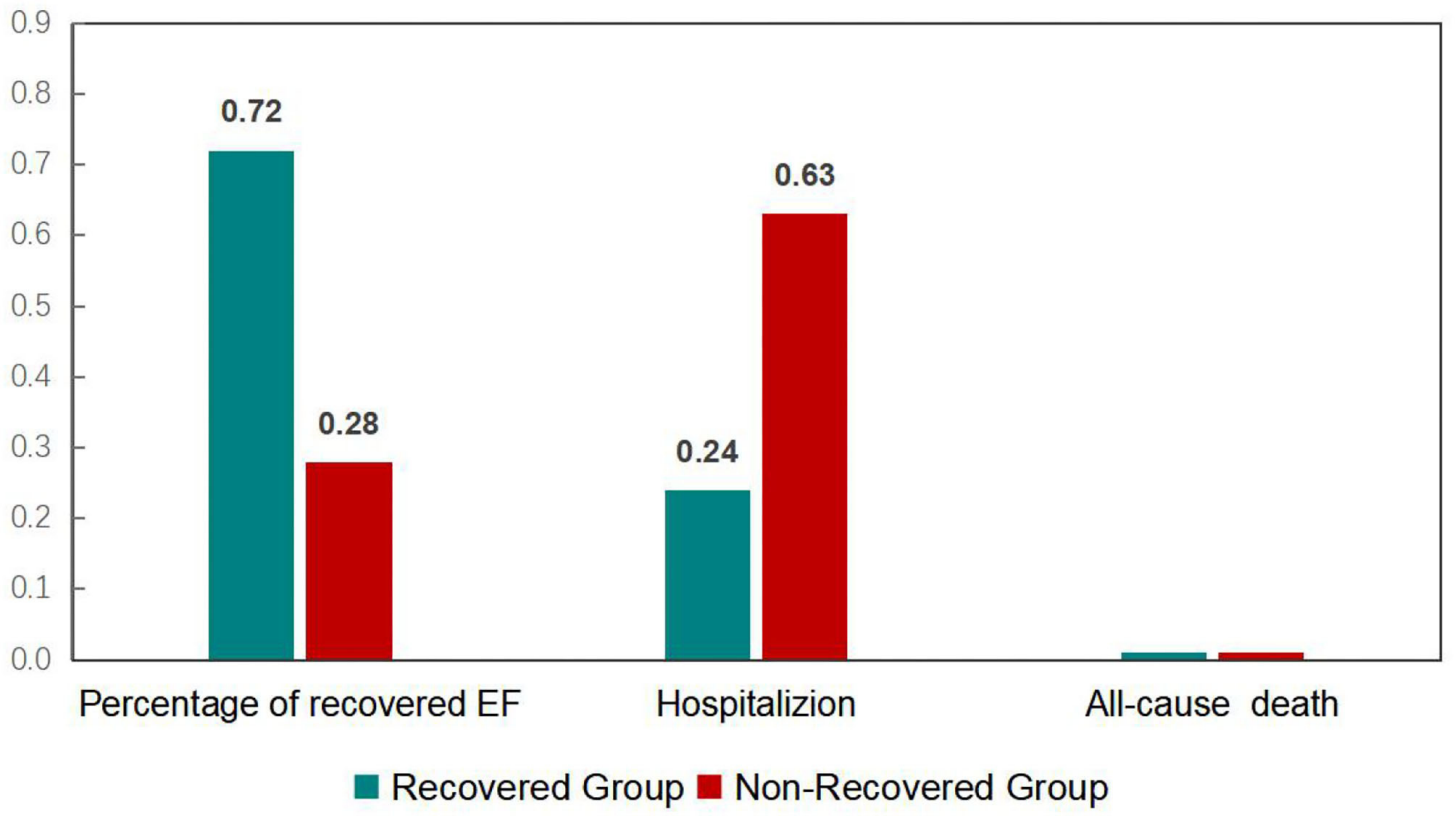
Recovery and outcome of heart failure patients undergoing atrial fibrillation after a one-year follow-up.

Left ventricular ejection fraction (LVEF) recovery occurred 1.5 ± 0.3 month after ablation was performed. The recovered and ablation outcomes are shown in Figure 1 and Table 2. The overall number of patients of LVEF recovered was 113 (72%). The mean LVEF was improved from 38% to 57% in the recovered group (*P* < 0.001), and the mean LVEF was not significant in the non-recovered group (from 0.36 to 0.38). After the 1-year follow-up, 25 (24%) hospitalizations occurred in the recovered group, and 27 (63%) hospitalizations occurred in the non-recovered group. The difference was significant [(odds ratio) OR 2.09 (1.40–3.12), *P* < 0.001]. No death occurred during the follow-up.

**Table 2:** Univariate analysis of predictors of recovered ejection fractions.

	**Recovered group (*n* = 113)**	**Non-recovered group (*n* = 43)**	**OR (95%CI)**	***P***
Age, mean (SD), years	62.8 (11.2)	65.9 (10.1)		0.175
**Sex, no. (%)**				
Male	77 (68.1)	28 (65.1)		0.356
Female	36 (31.9)	15 (34.9)		
**Type of AF, no. (%)**				
Paroxysmal	36 (31.9)	13 (30.2)		0.124
Persistent	77 (68.1)	30 (69.8)		
Duration of AF, mean (SD), years	3.1 (4.6)	3.9 (5.9)		0.427
Hypertension, no. (%)	53 (46.9)	20 (46.5)		0.965
Diabetes, no. (%)	24 (21.2)	4 (9.3)	0.38 (0.12–1.17)	0.083
Coronary disease, no. (%)	14 (12.4)	7 (16.3)		0.525
Valvular disease, no. (%)	10 (8.8)	6 (14.0)		0.348
CHA2DS2-VASc score, mean (SD)	2.4 (1.5)	2.3 (1.3)		0.914
LVEF, mean (SD), %	38.57 (8.0)	36.1 (6.8)	0.90 (0.91–1.00)	0.022
LAD, mean (SD), mm	43.0 (5,0)	43.9 (4.2)		0.278
LVD, mean (SD), mm	52.5 (6.8)	54.3 (6.8)		0.148
E/E′, mean (SD)	10.8 (4.2)	16.6 (9.5)	1.17 (1.08–1.26)	0.001
HR_max_, mean (SD), beats/min	140.5 (40.0)	140.8 (46.8)		0.972
HR_min_, mean (SD), beats/min	55.7 (11.5)	54.4 (14.8)		0.623
HR_mean_, mean (SD), beats/min	85.8 (16.9)	86.0 (20.7)		0.970
**Ablation Strategy, no. (%)**				
PVI only	53 (46.9)	20 (46.5)		0.913
PVI+line	48 (42.5)	18 (41.9)		0.827
PVI+CAFE	5 (4.4)	3 (7.0)		0.114
PVI+Line+CAFE	7 (6.2)	2 (4.6)		NS
Recurrence of AF, no. (%)	42 (37.2)	22 (51.2)		0.112
Prevalent AF, no. (%)	62 (54.9)	16 (37.2)	0.49 (0.24–1.00)	0.051
Prevalent HF, no. (%)	6 (5.3)	8 (18.6)	0.24 (0.08–0.76)	0.014
Concomitant AF and HF, no. (%)	45 (39.8)	18 (41.9)		0.817
All cause hospitalization, no. (%)	25 (23.8)	27 (62.8)	2.09 (1.40–3.42)	<0.001
All cause death, no. (%)	0 (0)	0 (0)	–	NS

*AF, atrial fibrillation; HF, heart failure; PVI, pulmonary vein isolation; CAFE, complex fractionated atrial electrograms; HR, heart rate; LVD, left ventricular diameter; LAD, left atrial diameter; LVEF, left ventricular ejection fraction; prevalent AF, existing AF and new-onset HF; prevalent HF, existing HF and new-onset AF; NS, no significant*.

### Predictors of LVEF Recovered

**Figure 2:**
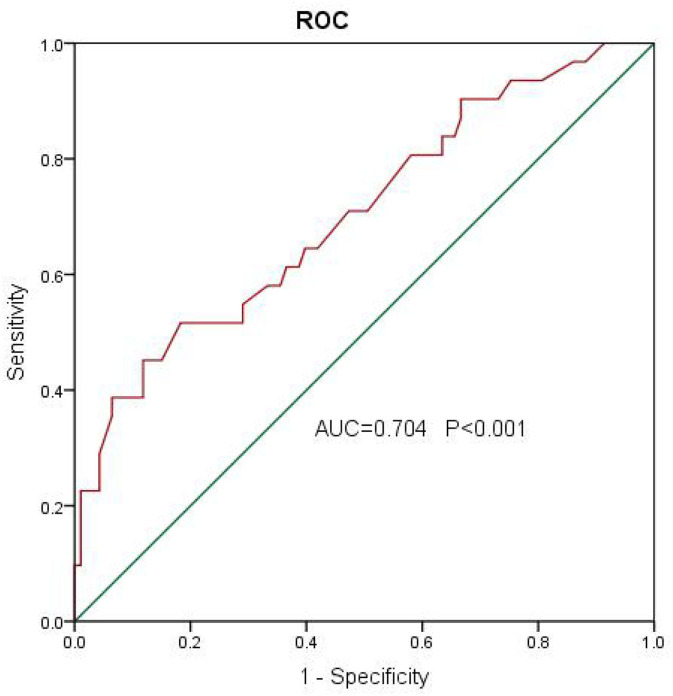
A receiver operating characteristic analysis revealed a moderate accuracy of predicting the improvement of left ventricular ejection fraction (LVEF) by E/E′ with a cut-off of 15 (sensitivity: 38.7%, specificity: 89.2%, area under the curve: 0.704).

We analyzed patients' baseline characteristics and procedural characteristics to evaluate the predictors of LVEF recovery (Tables 1, 2). Univariate analysis for recovered LVEF showed that diabetes (*P* = 0.083), prevalent HF (*P* = 0.014), prevalent AF (*P* = 0.051), LVEF (*P* = 0.022), and E/E′ (*P* = 0.001) were associated with LVEF improvement. Multivariate analysis showed that the only independent predictor of LVEF recovery was E/E′ [OR 1.13 (1.03–1.24); *P* = 0.011] (Table 3). A receiver operating characteristic analysis revealed a moderate accuracy of predicting the improvement of LVEF by E/E′ with a cut-off of 15 (sensitivity: 38.7%, specificity: 89.2%, area under the curve: 0.704) (Figure 2).

**Table 3:** Multivariate analysis of predictors of recovered ejection fractions.

**Examined parameters**	**OR (95% CI)**	***P***
Diabetes	0.23 (0.04–1.42)	0.115
Prevalent HF	0.35 (0.07–1.78)	0.205
Prevalent AF	0.45 (0.16–1.22)	0.115
LVEF	0.98 (0.92–1.05)	0.564
E/E′	1.13 (1.03–1.24)	0.011

*AF, atrial fibrillation; HF, heart failure; prevalent AF, existing AF and new-onset HF; prevalent HF, existing HF and new-onset AF*.

## Discussion

### Main Findings

Our main finding is that the only independent predictor of recovered LVEF is E/E′ < 15. In agreement with the literature, diabetes-prevalent HF, prevalent AF, and basic LVEF were significant predictors at the univariate level. Still, they were not independent predictors according to the multivariate analysis in our study. Therefore, this study may suggest a new predictor for LVEF recovered in systolic HF patients who receive AF ablation.

### The Outcome of Atrial Fibrillation Ablation in Systolic Heart Failure Patients

Since the early landmark study on AF ablation in congestive HF by the Bordeaux group, several observational and randomized studies have provided substantial evidence that AF ablation in patients with HFrEF results in high rates of successful sinus rhythm maintenance along with significant clinical and LV functional improvements (5). In the PABACHF trial (6), 76% of patients in the AF ablation group significantly improved LVEF vs. 25% in the atrioventricular nodal ablation/biventricular pacing group. In the CAMERA-MRI study (7), where only patients with persistent AF and idiopathic cardiomyopathy were included, 58% of patients had normalization of LVEF after AF ablation, compared with 9% of patients receiving medical rate control only. In our cohort of 156 consecutive cases, 72% of patients had recovered LVEF after AF ablation.

The most recently published larger prospective RCTs have evaluated the role and efficacy of AF ablation in selected patients with HFrEF. AF ablation was associated with a greater reduction in all-cause mortality and HF hospitalizations. In our study, recovered LVEF was associated with a 39% reduction in HF hospitalizations [23 vs. 63%; 2.09 (1.40–3.12), *P* < 0.001] compared to non-recovered LVEF. No deaths occurred in the study due to the small sample size and short follow-up. However, considering all RCTs together, it remains unanswered which AF ablation strategy is optimal and recommended to achieve the reported favorable outcomes. We compared the ablation strategy between the groups, and there was no difference in AF ablation for LVEF recovery. Advanced structural remodeling or non-convertible AF in HF patients following PVI (11) and additional ablation are often needed. Whether empirical linear lesions (12), targeted non-PV trigger ablation (13), ablation of complex fractionated atrial electrograms (CFAE) (14), or substrate (low voltage) modification (15) ensure superior success is a controversial matter of opinion, and comparison of the different ablation strategies in patients with HF lacks evidence.

### Predictors of Recovered LVEF

It should be considered in selected AF patients with HFrEF to improve survival and reduce HF hospitalization (IIa) following the 2020 ESC guidelines (16). We are aware that not every patient will benefit from an approach to ablation-based rhythm control. Thus, careful patient selection for AF ablation is key to achieving similar outcome benefits and should currently be geared to HF populations. Few studies have identified important factors that may be independent predictors of recovered LVEF after AF ablation (3). Ukita et al. (17) found that left ventricular end-diastolic dimension <53 mm might be an independent predictor of LVEF improvement after catheter ablation of persistent AF in HFrEF patients. In an academic review performed by Richter et al. (3), clinical guidance was proposed for the choice of treatment for AF in patients with HF, including ages <65, idiopathic cardiomyopathy, and LV diameter <55 mm. Studies suggest that a higher ventricular rate may be associated with an increased risk of HF in AF patients. However, in one study (18), among 50 patients with AF and HF who underwent AF ablation, only 15 (22%) had heart rate >100 beats per minute at baseline. However, LVEF normalized in 36 patients (72%) at 6 months post-ablation, suggesting that HF may develop in patients with no tachycardia.

We found that E/E′ is a new independent predictor of recovered ejection fraction in patients with systolic HF undergoing ablation for AF. Scarce data are available regarding the role and predictive value of E/E′ on the recovered ejection fraction. Our results suggested that E/E′ < 15 predicted an LVEF increase after AF ablation in patients with HF. E/E′ is the ratio between early mitral inflow velocity and mitral annular early diastolic velocity, which has become central in the guidelines for diastolic evaluation. The use of E/E' is generally the most feasible and among the most reproducible method for estimation of filling pressure. Several prominent validation studies have confirmed the correlation of this ratio with filling pressure, and the prediction of normal and abnormal filling pressure is most reliable when the ratio is <8 or >15 (19). Diastolic dysfunction is often associated with irreversible ventricular remodeling and poor outcomes (20). Higher E/E′ predicted more myocardial fibrosis (21), in addition to poor outcome after AF in HF. Patients with impaired diastolic and systolic functions suggest that cardiac reserve function is lost. Even if arrhythmias such as AF are corrected, cardiac function may not be restored. Concomitant systolic and diastolic dysfunction in patients with HF and AF may be a specialized form of cardiomyopathy and needs to be further assessed.

## Limitations

The major limitation of our registry is that it represents a single-center experience and the population size was underpowered. The follow-up TTE was performed at various times, which might have underestimated the improvement in LVEF. The “true” predictors of LVEF recovery were unclear. Further factors, such as genetics, atrial cardiomyopathy, tachycardiomyopathy, need to be studied. Our research is a retrospective study, and bias exists, such as patient selection, ablation strategy, etc.

## Conclusions

Left ventricle ejection fraction (LVEF) recovery occurred in 72.3% of patients, which was associated with a 39% reduction in all-cause hospitalization compared to the non-recovered LVEFs in our cohort. The only independent predictor of recovered LVEF was E/E′ < 15 in our series.

## Data Availability Statement

The original contributions presented in the study are included in the article/supplementary material, further inquiries can be directed to the corresponding authors.

## Ethics Statement

The studies involving human participants were reviewed and approved by Ethics Committee of First Affiliated Hospital of Dalian Medical University. The patients/participants provided their written informed consent to participate in this study.

## Author Contributions

YX, LG, and YaY contributed to conception and design of the study. MY, RZ, and HT organized the database. XG and YiY performed the statistical analysis. RZ and YS wrote the first draft of the manuscript. XX, XYu, YD, and XYi wrote sections of the manuscript. All authors contributed to manuscript revision, read, and approved the submitted version.

## Conflict of Interest

The authors declare that the research was conducted in the absence of any commercial or financial relationships that could be construed as a potential conflict of interest.

## Publisher's Note

All claims expressed in this article are solely those of the authors and do not necessarily represent those of their affiliated organizations, or those of the publisher, the editors and the reviewers. Any product that may be evaluated in this article, or claim that may be made by its manufacturer, is not guaranteed or endorsed by the publisher.
